# “It’s a Feeling That Makes You Do Anything”: Youth Narratives of Love and Experiences of Victimization in Their Romantic Relationships

**DOI:** 10.1177/07435584231190653

**Published:** 2023-08-02

**Authors:** Stéphanie Couture, Maude Lachapelle, Mylène Fernet, Martine Hébert

**Affiliations:** 1 Université du Québec à Montréal, Canada

**Keywords:** romantic relationships, violence, Canada, qualitative methods, technology/media/social media

## Abstract

Youth narratives of love are shaped by romantic experiences through observation of others’ romantic relationships, and by media commonly conveying romantic beliefs. Since past reports have linked romantic beliefs to dating violence (DV), studies need to explore narratives of love by youth who report DV victimization experiences to identify specific targets to address in DV prevention programs. This qualitative study explored the narratives of love by heterosexual youth and documented specific features according to their DV victimization experiences. Directed content analysis guided the analyses of semi-structured interviews of 82 participants aged 15 to 24 years (*M* = 19.4; *SD* = 2.1). Most participants were cisgender females (75.6%) born in Canada to Canadian-born parents (54.6%). Four polarized narratives of love emerged: (1) Growing love versus love at first sight, (2) Completive versus fusional love, (3) Lucid versus triumphant love, and (4) Ongoing versus eternal love. Both participants who reported experiencing DV victimization and those who did not expressed non-romantic and romantic beliefs, although they used different wording to convey similar beliefs in their narratives. These findings underscore the importance of challenging the dominant romantic beliefs that may place youth at risk of experiencing DV and therefore, contribute to DV prevention.

## Introduction

Adolescence is a crucial developmental stage during which youth usually seek more intimate contact with their peers, leading the majority to experience their first romantic and sexual relationships (Schleyer-Lindenmann & Piolat, 2011). Given their lack of experience, it may be particularly difficult for adolescents to figure out what love means to them while they are still in the process of shaping their own representations of love.

Throughout the years, research has generated multiple theories, operationalization, and measures of love (Soloski et al., 2013). Sternberg’s *Triangular Theory of Love*, for example, proposed three key components of what constitutes love: passion, intimacy, and commitment, with combinations of these aspects producing eight styles of love (Sternberg, 1986). Furthermore, Berscheid’s (2006) model suggested four types of love, namely companionate, compassionate, attachment and romantic/passionate love. Studies on love have primarily focused on quantitative data to assess these different components and have encountered significant limitations, such as inconsistencies in the administration of some scales due to the complexity of measuring distinguishable constructs about love. Thereby, the mobilization of qualitative methods appears essential to deepen the representations of love that can be reduced and decontextualized when examined exclusively via quantitative methods.

### Personal Conceptualization of Love and Its Sources of Influence

By redefining his initial theory, Sternberg (1998) recognized that people hold unique conceptions of love, which are the result of individual characteristics and messages extracted from the media, observations, and personal experiences. For instance, youth gather information about love and romantic relationships expectations by observing the relationships of their parents, discussing with their peers, and through exposure to mass media and sociocultural messages (de Lenne et al., 2020; Hefner & Wilson, 2013; Lippman et al., 2014; Sternberg, 1998). According to *Social Cognitive Theory*, socially accepted norms can be learned through the observation of role model behaviors and become internalized (Bandura, 2001), leading individuals to believe that their ideals are personal, for example, what love should be like (de Lenne et al., 2020).

Exposure to romantic beliefs through such sources may pressure adolescents to endorse romantic beliefs and lead to the internalization of idealized love by providing an unrealistic understanding of what love is (de Lenne et al., 2020). Romantic beliefs refer to the convictions surrounding love and romance that stipulate how an ideal romantic relationship should develop and function (Sprecher & Metts, 1989). Social and mainstream media, which most adolescents use for more than 5 hours per day (Katapally et al., 2018), are filled with examples of idealized romance (Lippman et al., 2014). Notably, in G-rated children’s films, the common representation of heterosexual romance portrays love as something powerful, magical, and transformative, and shows characters defying their culture, their parents, and even themselves to experience this love (Martin & Kazyak, 2009). Since romantic love is one of the most common representations of love in the media (Hefner & Wilson, 2013) and is as ubiquitous as technology, youth may incorporate these beliefs into their own definition of love and develop and experience their romantic relationships accordingly. In a qualitative study of 82 adolescents (*M*_age_ = 16.01), the written descriptions of what it means to be in love included five broad themes: commitment, intimacy, reciprocity, unconditional acceptance, and unsure/unknown (Williams & Hickle, 2010). The unsure/unknown component was used by adolescents to refer to their inability to describe love, either due to mystery or lack of experience, which may also reflect adolescents’ idealized views of love and their romantic inexperience.

Narratives of love may remain imbued with romantic beliefs in young adulthood. In a sample of 321 adolescents (*M*_age_ = 14.80), nonsignificant differences in the adherence to romantic myths between ages 11 to 14 and 15 to 18 were found; however, the belief that true love is predestined was found to be more prevalent in the older group (Bisquert-Bover et al., 2019). Previous studies have also shown that romantic beliefs do not differ according to age or number of past relationships in samples of young and older adults (Papp et al., 2017; Vannier & O’Sullivan, 2017), yet there is reason to believe that various experiences within romantic relationships may influence romantic beliefs. A longitudinal study found that adolescents who reported more dating violence (DV) victimization and perpetration showed more tolerant attitudes toward DV (Orpinas et al., 2013). In this regard, according to *Social Cognitive Theory*, youth self-evaluation of their personal romantic experiences may play an important role in reinforcing beliefs such as romantic beliefs acquired through environmental influences (Bandura, 2001). On the other hand, their self-reflective capacity may lead youth to judge the inadequacy of their romantic beliefs following personal experiences, thus allowing youth to modify their beliefs accordingly (Bandura, 2001).

### Impacts of Embracing Romantic Beliefs

Dominant in Western cultures, romantic beliefs include adherence to the ideology of romanticism (e.g., believing in love at first sight and one true love), the high value placed on romantic relationships, and the idea that jealousy is good (Papp et al., 2017). Media portray romance as heteronormative and frequently conflate love with jealousy, control, pain, tragedy, and violence (Hayes, 2014). As shown in a study of 270 young adults (*M*_age_ = 23.70) involved in dating relationships, the experience of romantic love can lead to positive outcomes, as romantic beliefs were associated with greater satisfaction and commitment to the relationship; however, idealization as an act of perceiving one’s partner through rose-colored glasses may be involved (Vannier & O’Sullivan, 2017). Romantic love can also be harmful when one’s feelings are unreciprocated and inappropriate, as shown by Fisher et al. (2016), who compared these experiences to substance or behavioral addictions. Engaging the same reward track of the brain, consequences of such negative addictions are substantial and can be extreme, as demonstrated by experiences of romantic jealousy that have led to homicide or suicide (Fisher et al., 2016). In a sample of 275 heterosexual women, romantic beliefs, such as misinterpreting controlling and jealous behaviors as signs of love, intimacy, and romance, were associated with romanticizing mate-retention behaviors (e.g., being contacted by your romantic partner to make sure you are where you said you were; Papp et al., 2017). It is through such behaviors, which are intended to prevent a partner from straying and are a means for one partner to dominate and control the other, that this study linked romantic beliefs to intimate partner violence victimization (Papp et al., 2017). In a sample of 217 women (*M*_age_ = 23.00), valuing jealousy in romantic relationships was related to normalizing violence in the relationship and forgiving a partner for perpetrating violent acts (Hartwell et al., 2015). Similarly, adults who endorsed stronger romantic beliefs were less likely to consider nonphysical violence in romantic relationships, such as coercive control behaviors, as abusive; these acts had to occur more often before being identified as such (Minto et al., 2022).

Previous studies have associated DV, which refers to any form of violence experienced by youth in current or past romantic relationships (i.e., physical, psychological, and sexual violence; Hébert et al., 2018), with romantic beliefs (Carrascosa et al., 2019; Cava et al., 2020; Lara & Gómez-Urrutia, 2021). More specifically, myths about romantic love or romantic beliefs were associated with peer aggressive behaviors, including offline and online DV victimization among adolescents (Carrascosa et al., 2019; Cava et al., 2020). In a study that also included young adults, the link with DV perpetration was also present (Lara & Gómez-Urrutia, 2021). A systematic review found varying prevalence of victimization in terms of physical DV (0.8%–32.9%), sexual DV (2.4%–41.0%), psychological/emotional DV (5.6%–95.5%), and technology-assisted DV (0.6%–48.0%; Tomaszewska & Schuster, 2021). A recent population-based study showed that one in three Canadian youth, in Grades 9 and 10 (*M*_age_ = 15.35 years) with dating experiences reported physical, psychological, and/or cyber DV victimization in the past 12 months (Exner-Cortens et al., 2021). Given its deleterious consequences (e.g., physical injury, emotional distress, depressive symptoms, and suicidal ideation; Hébert et al., 2018), DV is a serious public health problem that must be targeted by interventions. Given the established association between romantic beliefs and DV, it is essential to explore the narratives of love shared by youth experiencing DV victimization to help identify specific aspects of such beliefs that could be addressed and deconstructed through preventive interventions.

Love in the context of an abusive relationship is complex, according to a qualitative systematic review focusing on this phenomenon among women (Pocock et al., 2020). In abusive relationships, signals of intimate partner violence may be masked by the internal desire to romantically love and be loved. Women’s narratives mainly reported that power and control were signifiers of romantic love, which distinguished abusive and non-abusive relationships (Pocock et al., 2020). Hayes and Jeffries (2013), in a review of online social networking site discussion forums, also found that romantic love was the primary script for tolerating and enduring an abusive relationship and preventing women from leaving. In their study, many women who had experienced intimate partner violence believed that love conquers all and that if they truly loved their partner, forgave them, were patient enough, and “tried harder,” the violence would be prevented (Hayes & Jeffries, 2013, p. 67). Thus, the romantic belief that love conquers all may keep women trapped with their abuser, clinging to the hope that their partner will eventually change. Most studies exploring the interplay between violence and romantic love using a qualitative approach have focused on adult women, with less attention given to the experiences of youth. Given that adolescence and early adulthood are critical developmental periods during which youth experience their first romantic relationships and are exposed to romantic beliefs, it seems relevant to focus on their representations of love to act early to target and deconstruct most problematic romantic beliefs before they crystallize and become fully internalized.

### Current Study

Rather than being rooted in the researchers’ viewpoints, this study intended to provide a voice and a safe place for youth to open up about their representations of love and, for some, their DV victimization experiences. Considering that adherence to romantic beliefs can lead to the neglect or misinterpretation of partner behaviors, romantic beliefs provide a relevant analytical lens for understanding the ideologies underlying the acceptance of DV (Papp et al., 2017; Sprecher & Metts, 1989). The aims of the present study were to explore the representations of love of heterosexual adolescents and young adults, and to document specific qualitative features of the narratives of love for those with and without a history of DV victimization. More specifically, the research questions that this study sought to answer were:

RQ1. Which beliefs do youth endorse when defining love?
RQ2. How do youth who report DV victimization differ from their peers in terms of their narratives of love?

## Method

The present study used data drawn from a large mixed-methods study based on romantic and sexual experiences of adolescents and young adults. The data were collected from March 2015 to December 2017 in Quebec (Canada). The overall objective of the study was to explore the romantic and sexual experiences of adolescents and young adults, and to document communication and conflict resolution issues of youth with a history of victimization. The present analysis exclusively relied on the qualitative component of the study.

### Participants

Participants were recruited through an e-mail list of individuals from a previous study; the *Youths’ Romantic Relationships Project* (YRRP; Hébert et al., 2017), organizations from the Greater Montreal area, and surrounding places (e.g., schools, community organizations), by direct solicitations (e.g., flyer distribution, information booths) and through word of mouth. Research assistants screened potential participants for eligibility by telephone. To be eligible, participants had to be aged between 14 and 25 years and have had at least one sexual and one romantic relationship. To ensure homogeneity of the sample, partners living together or providing care for a dependent child were excluded. Also, this study focused on youth who identified as primarily heterosexual to ensure that youth experienced similar trajectories, since youth from sexual minorities may face different challenges in their romantic relationships.

Out of the 100 adolescents and young adults who participated in the interview, 82 participants addressed representations of love (*M*_age_ *=* 19.4; *SD*_age_ = 2.1). Most participants were cisgender females (75.6%), students (91.5%), born in Canada to Canadian-born parents (54.6%), and currently in a romantic relationship (62.2%) that had been going on for approximately one and a half years at the time of the study. On average, participants indicated having experienced 1.8 significant romantic relationships and reported having 5.8 sexual partners. Regarding DV victimization, 28.0% reported emotional or verbal violence, 25.6% reported physical violence, 24.4% reported sexual violence, and 13.4% reported threats. In total, approximately half of the participants (52.4%) reported at least one episode of DV in the last 12 months. Further detailed characteristics of the sample are presented in Table 1.

**Table 1.** Sample Characteristics and DV Victimization Experiences.

Characteristic	Total Sample (*n* = 82)	
	*M*	*SD*
Age	19.4	2.1
Number of significant romantic relationships	1.8	1.1
Number of sexual partners	5.8	6.7
	%	*n*
Gender		
Cisgender female	75.6	62
Cisgender male	24.4	20
Born in Canada to Canadian-born parents	54.6	45
Occupation		
Student, with or without work	91.5	75
Non-student worker	7.3	6
Neither student nor worker	1.2	1
Relationship status		
In a relationship	62.2	51
Single with a sexual partner	13.4	11
Single without a sexual partner	24.4	20
Dating victimization *(at least once in the past year)*	52.4	43
Emotional or verbal violence	28.0	23
Physical violence	25.6	21
Threats	13.4	11
Sexual violence	24.4	20

### Procedures

After completing an online questionnaire as part of the larger study, participants were invited to participate in an individual semi-structured interview, which lasted 75 min on average. The interview covered themes such as: dating and sexual experiences, communication, and conflict management, dating violence, and help-seeking strategies. Participants completed the interview with a research assistant face-to-face at our laboratory, in a public place near their home (e.g., private room in a library or a college), or online via a video conference. Prior to the interview, the research assistants, who were trained to intervene in crisis situations, clearly explained objectives, confidentiality measures, procedures, benefits, and risks of the study and signed the consent form with the participants. Adolescents were allowed to participate in the study without parental consent, as the established age of consent in Quebec (Canada) is 14 years. Given the potential risks in terms of discomfort and distress, the following measures were implemented: (1) youth were informed of the potential risks of the study; (2) negative emotions were monitored throughout the data collection process; (3) a list of resources was offered to participants (e.g., phone hotlines, websites) following their participation, with guaranteed follow-up; and (4) a protocol was planned to be implemented in the event of a crisis (which did not occur). All participants received $25 CAD as financial compensation. This study was approved by the institutional research ethics board of the authors’ affiliated University (#1613_e_2017).

### Analysis

With the participants’ consent, interviews were audio recorded and transcribed verbatim. To meet the aims of the present study, the following question was analyzed: Can you describe what love means to you? This question was general enough to allow youth to respond freely, and for romantic and non-romantic beliefs to emerge from their responses. Given the objectives of our study, directed content analysis was the preferred method of assessment to allow for a theory-guided analysis. Inspired by a 16-step data analysis method of directed qualitative content analysis (Assarroudi et al., 2018), several operations were carried out to complete this analysis. A careful reading of the verbatim to get an overview of the data was accomplished. A coding procedure, which allowed the entire verbatim to be divided into units of meaning (i.e., a set of sentences related to the same idea; Tesch, 1990) and labeled using a coding grid, was implemented. Codification was performed using a mixed coding grid developed in part beforehand according to a framework in which other codes were added in light of the empirical material analyzed. The initial coding grid was inspired by the dimensions of the *Romantic Beliefs Scale* (Sprecher & Metts, 1989), the most widely used instrument in research on romanticism and relationships (Lippman et al., 2014). This instrument captures romantic beliefs through four subscales, each of which expresses a facet of romanticism in relationships: (1) *Love Conquers All* (i.e., the belief that love can overcome all obstacles); (2) *One and Only* (i.e., the belief that we have a soul mate); (3) *Idealization* (i.e., the belief that true love is perfect); and (4) *Love at First Sight* (i.e., the belief that we know it immediately when we meet the right person). Thus, each of these subscales refers to a different facet of the relationship, that is, (1) the obstacles encountered within the relationship, (2) the duration of the relationship, (3) the relationship dynamics, (4) the emergence of romantic feelings. The mobilization of this theoretical framework allowed us to have facets in mind during the analysis, which allowed the identification of romantic beliefs to emerge in addition to non-romantic beliefs. Once the coding was completed, an output of the codes and accompanying excerpts was generated to facilitate the next stage of analysis. Then, categorization allowed us to qualify the units of meaning identified to better understand the phenomenon under study (Tesch, 1990). This procedure consisted of forming mutually exclusive conceptual categories by grouping excerpts dealing with the same concept. Conceptual categories were developed and then linked together to identify the thread that connected all of the categories to develop a coherent story that reflected the participants’ voices. Finally, the features of the representations of love within the identified conceptual categories were explored to highlight the similarities and divergences of those with and without a history of DV victimization. To this end, the specific qualitative features of the narratives of participants who reported DV victimization experiences and those who did not were investigated within categories (i.e., the nuances between what was reported by each group), but also between categories (i.e., which categories were reported predominantly or exclusively by participants within a group). Some participants spontaneously addressed DV victimization experiences following the question: What are the worst difficulties you have encountered in your relationships? In addition, youth were asked about their knowledge of DV manifestations before being asked about their experiences with any of the identified manifestations (i.e., How do you think violence can manifest in a romantic relationship? Have you ever experienced any of the manifestations of violence you mentioned in your romantic relationships? If so, which one(s)?). Finally, we confirmed the presence of a history of DV victimization using the Conflicts in Adolescent Dating Relationships Inventory (CADRI; Fernandez-Gonzalez et al., 2012), since each participant had also completed a self-reported questionnaire. When participants reported DV victimization experiences, it was indicated following the quote with an assigned fictitious name and the age of the participant. To ensure the reliability of the results, three researchers reviewed the analyses throughout the process (Noble & Smith, 2015). A team coding procedure (Weston et al., 2001) was used, and team meetings were held to ensure a common understanding of the coding grid, to discuss and refine emergent codes, and to debate certain parts of verbatim that were more difficult to code. One researcher then initiated the categorization, and two researchers strengthened the analysis by revising it several times; discrepancies were discussed to reach a consensus. The NVivo12 software was used to support this analysis (QSR International Pty Ltd, 2018).

## Results

The purpose of this analysis was to explore representations of love of heterosexual adolescents and young adults, and to identify specific features within the narratives of those who reported DV victimization experiences and those who did not. The analysis yielded four conceptual categories that reflected divided opinions on each of the facets of love outlined in the method section. To address the first research question, these divided opinions were presented in different subcategories within each category to better differentiate non-romantic beliefs that youth endorse when defining love from romantic beliefs. To answer the second research question, in each of the subcategories, we indicated it when the results were reported only by participants who reported DV victimization or by participants who did not report DV. The first number in brackets following the names of subcategories refers to the number of participants out of the total sample endorsing the described narratives of love, while the latter refers to the number of participants who reported DV victimization experiences among them.

### Growing Love Versus Love at First Sight: Emergence of Romantic Feelings

Participants developed their perception regarding the emergence of romantic feelings. Some participants considered that feelings evolve gradually, while others perceived love as something that must be felt from the very beginning.

#### Growing Love: Feelings That Develop Over Time (13, 6DV)

Participants adopted a similar discourse, regardless of their DV victimization experiences, when discussing the gradual evolution of feelings toward love. Participants mentioned that love is a process that goes through friendship before becoming lovers. Some participants revealed that the distinction is primarily one of sexual attraction, as the foundations of friendship are amplified by sexual tension and desire: “I didn’t find a huge difference between the deep friendship we had before and now, except for the sexual and deeper intimacy, but before he was also someone with whom I liked to share my small successes and vulnerabilities.” (Jade, 21 years old). Few participants reported this transition as a time-consuming process achieved by getting to know each other, developing mutual trust, and finding common interests. They described love as something you have to invest yourself in if you want it to grow: “I may not have wanted to invest more so it stopped. I don’t know if I took the time. [. . .] I didn’t take the time to fall in love. I think if it had continued, it would have turned into something.” (Gabrielle, 20 years old). Participants in this subcategory mentioned they did not believe in love at first sight and the feeling of having butterflies in their stomach. They perceived these romantic beliefs as not truly related to love itself, which they conceived as a much more complex process to understand; a process that develops daily through small acts: “I don’t think it’s the butterflies or the super strong emotions that you might feel. [. . .] I don’t believe in butterflies. It’s more the daily routine, the small things that we do, the little moments.” (Coco, 20 years old, DV victimization).

#### Love at First Sight: Feelings Obvious From the Beginning (8, 5DV)

Other participants discussed feelings of love that settled down more quickly. All participants in this subcategory revealed love was obvious to them from the beginning.

Participants who did not report DV experiences described the physiological sensations they felt when they first saw their loved one, such as butterflies in their stomach and other signs related to stress or great joy: “When you see that person, you have butterflies in your stomach. You just see their name on your phone and you’re all cheerful like you’re eating a chocolate cake as a child. [laugh]” (Ashley, 20 years old). Participants who reported DV experiences, however, mentioned experiencing love at first sight without describing the sensations they felt in their bodies. They admitted that they did not know how to explain this phenomenon and did not refer to what this experience meant in terms of physiological sensations: “I had a bit of an experience of love at first sight. I saw him and I felt like I had to get to know him. [. . .] That’s love. I don’t really know how to explain it. It’s special.” (Katya, 16 years old, DV victimization).

### Completive Love Versus Fusional Love: Relationship Dynamics

Participants opened up about love regarding the relationship dynamics. Some considered love as a complement to oneself, while others perceived love as necessary to feel like a whole person.

#### Completive Love: A Partner Who Contributes to Personal Fulfillment (25, 9DV)

Many participants described love as an opportunity for self-actualization. They described love as offering the potential to become a better person by perceiving the partner as someone who encourages and pushes them to surpass themselves as individuals: “It’s something that pushes you to be a better person and brings out the best in you [. . .]. Love is about encouraging, accepting, and bringing out the best in each other. It’s not just one side.” (Eve, 20 years old). Participants expressed that they value mutuality and expect their partner to be supportive and to provide a safe space to share personal experiences. They identified several components that were important to them in a romantic relationship, including commitment, communication, respect, trust, and cooperation, and some described a couple as a team: “A couple is trust and communication. I think it all comes together. It’s a team. The more you trust the person, the more you will communicate your concerns or what you feel.” (Jasmine, 21 years old, DV victimization). Several participants discussed the importance of considering their partner in their own decisions and personal plans. Caring and considering for each other were viewed as gestures of love.

Participants who did not report DV experiences emphasized that the other’s consideration should not be done at one’s own expense and that no one should forget their own individuality when it comes to love: “There is also self-love, you want the best for the other person, but never at the expense of yourself. The old saying that claims that before loving others you need to love yourself; I deeply believe it.” (Prudence, 21 years old). Also, participants who did not report DV experiences viewed love as something that pushes you to be more of the person you are meant to be: “It’s like a puzzle that allows you to see the picture, but two or three pieces are missing. This person comes and puts her pieces, it doesn’t make you another puzzle, you’re just more who you’re supposed to be.” (Mila, 19 years old).

#### Fusional Love: An Idealization of Love, the Relationship, and the Partner (11, 9DV)

Among the participants who described love as a fusional experience, the majority reported DV victimization experiences. Participants in this subcategory reported a strong interdependence between partners, to the extent of endorsing their values, decisions, and daily experiences as their own. Some participants even claimed that the partners eventually belong to each other or that they become one: “When it’s love, the real, [. . .] it’s a little cliché but you become a person together, then it’s the only one. I think she’s the only person that really matter.” (William, 19 years old). Few participants recognized that love led them to prioritize their partners over themselves and their own needs and desires. They said they gave up their own interests out of love and were willing to sacrifice everything for their loved one: “You’re going to make sacrifices, like you want a house with him, you are not going to buy a motorcycle [. . .]. You are willing to sacrifice everything for this person because you think she’s the one.” (Charlotte, 17 years old, DV victimization).

Participants who reported DV experiences shared that love led them to idealize their partners, perceiving them as unique, perfect, and flawless: “When you have more feelings that come into play [. . .] at the beginning you see that the person has no flaws, you’re like “ah she’s so beautiful and everything”, I like her flaws.” (Karianne, 17 years old, DV victimization).

### Lucid Love Versus Triumphant Love: Obstacles Encountered Within the Relationships

Participants discussed love in regard to the obstacles encountered in the relationship. Some considered love as having limits, while others perceived it as overcoming everything.

#### Lucid Love: Don’t Accept Everything Out of Love (27, 13DV)

Many participants described love as an experience where some boundaries should not be crossed. Participants perceived mutual involvement in conflict resolution as a key to navigate through challenging times. They discussed the importance of being patient, accepting their partner for who they are without necessarily agreeing with everything they do, and communicating to maintain the relationship: “We almost split up and finally we saw each other [. . .]. He advocated for communication, as I did too. We talked to each other, we got over our differences and now our anniversary is the 2nd of the month.” (Prudence, 21 years old). For some, the absence of violence was a criterion for establishing a romantic relationship, and the family education received had anchored the importance of being treated respectfully and nonviolently: “He’s not supposed to do this to me. My mom always told me never to let a guy hit me because I don’t deserve it. Like, that’s not how a guy who loves you is supposed to treat you.” (Katya, 16 years old, DV victimization).

Some participants who reported DV experiences admitted that they were seeking a violence-free relationship as a result of their previous DV experiences or after witnessing their peers or their parents being abused, highlighting the role of relationship models in their representations of love. They also emphasized that they will no longer accept what they once tolerated and that they have become more able to recognize that abusive behaviors are unacceptable. Participants who did not report DV experiences discussed in this subcategory love by addressing the importance of feeling comfortable, respecting their boundaries (e.g., not allowing insults even as a joke), and leaving when your partner oversteps your limits: “As a lover, love would be. . . not too unconditional, because you have to respect your limits and try to understand what’s going on yes, but like knowing when to say no, and then leave when you have to.” (Veronique, 21 years old).

#### Triumphant Love: True Love Can Overcome All Obstacles (20, 13DV)

Other participants described love as a feeling that can overcome all obstacles. Participants described love as a feeling that can triumph over everything, as something that gets you through difficult times, and allows people to stay together despite adversity. Some participants in this subcategory, whether or not they experienced DV victimization experiences, reported that love was about being willing to do anything for each other: “Love is really strong, it’s a feeling that makes you do anything, that makes you think. . . that makes you completely paranoid, in my opinion, sometimes.” (Tammy, 21 years old, DV victimization).

Participants’ narratives in this subcategory showed cues that minimize violent behaviors in their romantic relationships. Of all those who did not report DV experiences, only one participant revealed such signs by normalizing the use of verbal abuse as name-calling in a relationship when they were truly angry. As for participants who reported DV victimization, they minimized the abuse they endured by stating that they thought some forms of violence were more acceptable than others and that their victimization experiences were not serious: “It’s always been couple stuff, it’s not. . . we’ve never had a fight. We never went to that extent. Often a slap, push, kind of basic stuff.” (Elea, 20 years old, DV victimization).

### Ongoing Love Versus Eternal Love: Duration of the Relationship

Participants discussed the duration of the relationship. Some considered love as a feeling that changes over time, while others as a feeling that lasts forever.

#### Ongoing Love: Loving One Day at a Time (16, 12DV)

Some participants described love as a feeling that fluctuates, stating that over time, love changes and may eventually fade: “Love for me is a feeling of attachment, which is very beautiful, but eventually fades. In the end, it’s more a decision to love someone than really the feeling of attachment that you feel continuously. Love is a choice.” (Vanessa, 21 years old). Some participants reported that they prefer to live in the moment rather than project themselves into the future. They emphasized how this allowed them to let life take its course and to get rid of the expectations related to the future: “There’s always going to be the possibility of hitting a wall and not working out. We leave a lot to chance, and we free ourselves from the burden of expectations of the future. We live in the present.” (Adam, 22 years old, DV victimization).

Only participants who did report DV directly challenged in their narratives the concept of true love, the popular myth of romantic love. Participants reported not believing that there is only one person they can make a life with, nor that love is a feeling that can only be felt once: “It’s a kind of chemistry between two individuals. Maybe it’s not unique. I think it’s possible to love more than one person in a lifetime. I don’t know how to describe it.” (Danielle, 19 years old, DV victimization). One participant explained that this questioning was triggered by a love deception that changed his beliefs and expectations about love, as he had previously thought that he would follow his mother’s example and spend his whole life with the same person: “I wanted to do like my mom, have just one partner and like wow. But that girl just kind of used me. So then, you know, I didn’t believe that anymore. I was like well there, fuck off.” (Sebas, 19 years old, DV victimization).

#### Eternal Love: Loving Each Other Forever (16, 8DV)

Other participants described love as a long-lasting feeling and mentioned that their partner had to be someone with whom they could see themselves making plans and spending their whole life: “When you’re in love you want to stay with that person all your life and you’re going to have kids, have projects together that you want to do just together and not with a bunch of friends.” (Catherine, 20 years old, DV victimization). Both participants who reported DV experiences and those who did not in this subcategory seemed to embrace the romantic belief that there is one and only love, using terms such as the *right person*, the *love of their life*, and their *other half.* Few participants acknowledged that they had no choice but to be with their partner. They mentioned they could not imagine themselves without their partner and that they would not want to lose their partner for anything: “Love is feeling like you can’t let go of a person. You will always be there for each other. You will never accept to see them fall. I couldn’t imagine living without him. That’s what love is to me.” (Jessica, 21 years old).

## Discussion

This study offers new insights into representations of love from the perspective of heterosexual adolescents and young adults. The *Romantic Beliefs Scale* (Sprecher & Metts, 1989) facilitated the identification of these individuals’ love-related beliefs in their narratives, thereby enabling the emergence of distinct conceptual categories encompassing a comprehensive spectrum of what love means to them. Four categories emerged from which participants reflected varied representations of love pertaining to different facets of their relationships, including the emergence of romantic feelings, relationship dynamics, obstacles encountered within the relationship, and the duration of the relationship.

### Beliefs Endorsed by Youth When Defining Love

Although romantic beliefs are common among youth, most participants offered a variety of perspectives on love that differed among the facets of love represented within the emerging categories, providing examples of romantic and non-romantic beliefs. Except for the last category, in general, a larger number of participants fell into the subcategory depicting non-romantic beliefs. In the first category, five more participants evoked the gradual emergence of romantic feelings from friendship than a perception that such feelings appeared more suddenly, akin to love at first sight. Concerning the second category, narratives highlighting the belief that a romantic partner should contribute to one’s self-fulfillment were reported twice as much as love as fusional. Within the third category, seven more participants referred to love as having certain limits than to love as transcending every obstacle encountered within the relationship. Finally, in the fourth category, beliefs encompassing love as an evolving and fluctuating feeling were as frequently reported as beliefs surrounding love as a feeling that lasts forever. Also, it is worth noting that participants themselves acknowledged changes in their representations of love resulting from their personal experiences. These changes were not limited to instances of DV victimization; they were observed more broadly following disappointments in their romantic relationships.

### DV Victimization and Narratives of Love

Both participants who reported DV victimization experiences and those who did not shared narratives that tended to be imbued with non-romantic beliefs and romantic beliefs, such as love at first sight and one true love. Although we did not observe differences between categories based on DV victimization experiences, specific qualitative features emerged within categories in the participants’ narratives, revealing that youth who reported DV victimization experiences as well as those who did not, used different wording to express similar beliefs. This finding is inconsistent with previous studies linking romantic beliefs and DV (Carrascosa et al., 2019; Lelaurain et al., 2021), as we would expect that participants with a history of DV victimization experiences would display more romantic beliefs than those who did not.

Regarding qualitative features according to DV experiences in the first category, only three individuals in the sample spontaneously expressed the physiological sensations experienced during the emergence of romantic feelings, and these were participants who did not report DV experiences. Participants reporting DV experiences may experience disembodiment following their experiences, in a way that makes them unaware of their deep feelings and have a numbed awareness of their internal states when attempting to describe love. This interpretation is consistent with previous studies focusing on adult survivors of intimate partner violence, indicating that they can disconnect from their body and/or emotions in order to protect themselves from the abuse and the associated consequences (Matheson et al., 2015; Sinko & Saint Arnault, 2020).

Regarding the second category, among the 25 participants revealing completive love beliefs, only a subset of participants who did not report DV victimization expressed the idea of maintaining self-identity while loving another individual. In contrast, the majority of participants who embraced fusional love beliefs (i.e., 9 out of the 11) reported being victims of DV. Also, although youth in the latter subcategory were the only ones to idealize their partners, the two participants who did not report DV instead idealized love in general or their own relationship. These findings in the narratives may reflect the well-documented association in the literature between DV victimization and an anxious attachment style (Bonache et al., 2017), characterized by traits such as dependency, fusion, excessive reliance on others, positive evaluation of others, and negative self-evaluation (Ross et al., 2016). The narratives of love of adolescents and young adults reporting DV victimization exhibited a greater presence of these traits. The act of idealizing one’s partner may place youth at a heightened risk of being subjected to their partner’s needs or control, thereby potentially becoming a risk factor for experiencing DV.

Furthermore, of all participants with lucid love narratives, approximately half had experienced DV victimization (i.e., 13 out of 27), and few of these participants acknowledged changes in their beliefs (e.g., absence of violence became a criterion for future relationships) after experiencing DV themselves or witnessing violence between their parents or friends. In line with the posttraumatic growth framework, cognitive perspectives have the potential to undergo positive changes and development following trauma, as individuals engage in a cognitive struggle amidst adverse life circumstances (Tedeschi & Calhoun, 2004). Typically, these cognitive shifts commonly occur through social interactions rather than independent self-discovery. Some survivors of DV may experience such transformative growth by engaging in healthier relationships, where they learn that love can be expressed and received without enduring physical or emotional harm (Dagan & Yager, 2019). Some of the participants who reported DV experiences in the present study may have restructured their representations of love due to various adverse life events or as a result of encountering a positive romantic relationship for the first time. Moreover, out of the 20 participants endorsing triumphant love beliefs, while only one participant who did not report DV exhibited in their narratives a tendency to downplay the significance of violence in romantic relationships, several participants who reported DV victimization displayed this attitude. This finding implies that the conviction that “love conquers all” may introduce potential risks, particularly in the romantic relationships of adolescents and young adults who have endured DV and may still be involved in a relationship with their abuser. This belief can foster hope for change in an abusive partner’s behavior, consequently entrapping individuals with their perpetrator (Papp et al., 2017).

Finally, of the 16 participants in the ongoing love subcategory, all of those who directly contested the concept of true love (i.e., there is only one person to live with and love forever) reported DV victimization experiences. This finding may reflect the evolution of beliefs following personal experiences (e.g., DV victimization) or, according to *Social Cognitive Theory*, the self-reflective capacity of individuals regarding the inadequacy of their initial romantic beliefs (Bandura, 2001). Our results are consistent with prior research suggesting that DV experiences can alter participants’ perception of their relationship (Brosi & Rolling, 2010), which in turn may lead them to confront their initial representations of love with disillusionment. In fact, the inability to find the actual experience of the expected form of love can result in disillusionment, which refers to the decline in positive perceptions and can be a consequence of initial idealized perceptions (Niehuis et al., 2011). DV victimization may lead some adolescents and young adults to become disillusioned with love more quickly than others who did not report DV experiences. Although this interpretation is consistent with the results of the current study, future research should examine this hypothesis.

### Limitations and Strengths

A few limits must be considered to appreciate the findings of the present study. First, although this study mobilized a substantial sample size for a qualitative study, our sample of only heterosexual, mostly female student participants may have shaped the categories that emerged. Further research should look beyond this homogeneous sample to document youth narratives of love. Second, despite the fact that representations of love were implicitly addressed by the participants, the reliance on a single explicit question to explore participants’ narratives of love may have limited the exhaustiveness of the results. A semi-structured interview with questions that focus on the different facets of love and on romantic and non-romantic love-related beliefs would help identify more nuances in narratives of youth. Third, some participants had conflicting narratives of love as they differentiated their current representation from the one they had when they were younger. Although it is natural for youth to experience changes in their beliefs, future studies should examine the evolution of romantic beliefs, using a longitudinal design or a methodological approach relying on a timeline or life history calendar, to explore the trajectory and changes in youth narratives of love and the different milestones that led to these changes. Fourth, the current study did not consider when participants experienced DV and whether or not they were still engaged in a relationship in which violence occurred. Future studies should take these factors into consideration and explore the differences between the narratives of love of youth who are still involved in a violent relationship versus those who have left such dynamics. Finally, the analysis focused solely on the reported experience of DV victimization, or lack thereof, when exploring romantic and non-romantic beliefs in participants’ narratives. However, it is important to acknowledge that additional factors may contribute to our understanding of youth representations of love. For instance, one study found that although age was not directly associated with romantic beliefs, the effects of watching romantic movies decreased with increasing age during adolescence (Driesmans et al., 2016). Moreover, considering the severity and forms of DV victimization may be relevant given the associations between romantic beliefs and minimizing some forms of violence (e.g., nonphysical) but not others (Minto et al., 2022). Future studies should consider these potentially important predictors and moderators when investigating DV victimization experiences and adherence to romantic beliefs.

This study allowed us to provide a safe space for youth to open up about their narratives of love and to explore the nuances within the discourse for those with and without a history of DV victimization. In addition, the study exploited different facets of relationships in the representations of love, resulting in a more holistic insight into adolescents’ and young adults’ perceptions. Even though representations of love are imbued with a great deal of subjectivity, many studies that focus on romantic beliefs have used quantitative method almost exclusively, rather than investigating the meaning of youth perceptions. In this sense, one of the greatest strengths of this study was its focus on adolescents’ and young adults’ narratives from their perspective in order to inform and target the needs in terms of intervention as expressed by youth themselves.

### Implications for Practice

The findings of the present study offer relevant implications for educational and therapeutic interventions. First and foremost, before talking about violence with adolescents and young adults, it is important to talk about love in a way that does not perpetuate romantic beliefs and to talk about positive romantic relationships so that youth are able to recognize and engage in them. Given that few participants who reported DV victimization experiences showed acceptance of some forms of violence, intervention programs should be implemented in early adolescence and help youth recognize the different forms of violence, as some do not consider certain behaviors as abusive because they are not severe enough. Interventions should also target a shift in narratives by dismantling beliefs such as “love conquers all,” perceiving one’s partner as perfect and flawless, and interpreting a partner’s violent behaviors as a sign of love or as something insignificant, all while promoting non-romantic beliefs. These types of interventions could bring positive outcomes for youth and their relationships and help them recognize violent behaviors as controlling and abusive. Also, considering the wide range of romantic beliefs discussed by the participants in our study, future interventions should promote the growth of a critical perspective in adolescents and young adults to understand the influence of how love is portrayed through social norms and media. By doing so, youth may be better equipped to understand how these social influences affect their behaviors and attitudes in their romantic relationships and to develop personal and significant representations by recognizing their own beliefs. Challenging and deconstructing the dominant romantic discourse on love appears to be particularly promising as a target for future DV prevention programs (Lelaurain et al., 2021; Ustunel, 2020). Moreover, professionals should support youth in reflecting on their past romantic and DV experiences and on how these experiences impact their narratives of love. In addition to developing critical thinking skills, interventions involving youth who report DV victimization should foster the development of self-esteem to promote youth’s autonomy and lead them to understand that their own needs are equally important as their partner’s. Similarly, interventions should focus on reconnecting with oneself, one’s own sensations and emotions, and building motivation and self-belief to establish and maintain positive and healthy romantic relationships after DV victimization.
